# Comparative Effectiveness of Bevacizumab and Olaparib Maintenance Therapy in Platinum-Sensitive Recurrent Ovarian Cancer: A Real-World Study with Exploratory Evaluation of Dose Reduction

**DOI:** 10.3390/cancers18091332

**Published:** 2026-04-22

**Authors:** Shunsuke Tatsuki, Tadahiro Shoji, Ami Jo, Nanako Jonai, Yohei Chiba, Sho Sato, Eriko Takatori, Yoshitaka Kaido, Takayuki Nagasawa, Masahiro Kagabu, Takeshi Aida, Fumiharu Miura, Tsukasa Baba

**Affiliations:** 1 Department of Obstetrics and Gynecology, Iwate Medical University School of Medicine, Yahaba 028-3695, Japan; tatsuki-s12@outlook.jp (S.T.); amipc7@gmail.com (A.J.); jonain@iwate-med.ac.jp (N.J.); youcfaith51@yahoo.co.jp (Y.C.); satos@iwate-med.ac.jp (S.S.); takatori@iwate-med.ac.jp (E.T.); ykaido@iwate-med.ac.jp (Y.K.); tnagasaw@iwate-med.ac.jp (T.N.); mkagabu@iwate-med.ac.jp (M.K.); babatsu@iwate-med.ac.jp (T.B.); 2 Department of Obstetrics and Gynecology, Hachinohe Red Cross Hospital, Aomori 039-1104, Japan; t-aida.ob-gy@nifty.com; 3 Department of Obstetrics and Gynecology, Iwate Prefectural Central Hospital, Morioka 020-0066, Japan; mfumiharu@gmail.com

**Keywords:** ovarian cancer, platinum-sensitive recurrence, maintenance therapy, bevacizumab, olaparib

## Abstract

**Objective**: To compare real-world PFS between BEV and OLA as maintenance therapy for PSROC, with an exploratory evaluation of clinical outcomes after OLA dose reduction. **Methods**: This retrospective multicenter study included 101 patients with first platinum-sensitive recurrent ovarian, fallopian tube, or primary peritoneal cancer who achieved a response to platinum-based chemotherapy and then received maintenance therapy. Patients were classified into three groups: BEV (n = 34), standard-dose OLA (n = 31), and dose-reduced OLA (n = 36). The primary endpoint was PFS; secondary endpoints were OS and adverse events. Survival outcomes were evaluated using Kaplan–Meier methods and Cox proportional hazards models. **Results**: In the primary comparison of all OLA-treated patients versus BEV, OLA was associated with longer PFS (HR 0.48, 95% CI 0.29–0.77), with median PFS of 19 months versus 16 months, respectively. OS did not differ significantly between groups (HR 0.60, 95% CI 0.34–1.05). In exploratory subgroup analyses, patients who underwent OLA dose reduction had numerically longer PFS than those who remained on the full dose; however, this comparison is vulnerable to time-dependent and selection biases and should be interpreted cautiously. Grade ≥ 3 hematologic toxicities were more frequent in the OLA groups but were clinically manageable. **Conclusions**: In real-world practice, OLA was associated with longer PFS than BEV in PSROC. Clinically necessary dose reduction appeared feasible without an obvious loss of benefit, although this finding requires cautious interpretation.

## Simple Summary

In platinum-sensitive recurrent ovarian cancer (PSROC), bevacizumab (BEV) and olaparib (OLA) are commonly used maintenance options after response to platinum-based chemotherapy. However, direct real-world comparisons in biomarker-heterogeneous populations are limited, and the clinical significance of OLA dose reduction remains unclear. In this multicenter retrospective study of 101 patients, the primary analysis compared BEV with all OLA-treated patients, whereas analyses according to OLA dose intensity were exploratory. OLA was associated with longer progression-free survival (PFS) than BEV, while overall survival (OS) did not differ significantly. Because treatment allocation was non-randomized and the dose-reduction analyses are susceptible to selection and time-dependent biases, these findings should be interpreted cautiously. Nonetheless, clinically necessary OLA dose reduction appeared feasible and was not associated with an obvious loss of benefit in selected patients in routine practice.

## 1. Introduction

Ovarian cancer is one of the most lethal gynecologic malignancies and is frequently diagnosed at an advanced stage, with a clinical course characterized by repeated recurrences [1]. The incidence of ovarian cancer is reported to be approximately 1.3 times higher than that reported a decade ago [2]. In 2020, approximately 314,000 new cases of ovarian cancer and 207,000 related deaths were reported worldwide [3]. In the United States, an estimated 19,680 new cases and 12,740 deaths from ovarian cancer were predicted in 2024 with mortality from ovarian cancer exceeding that from cervical and endometrial cancers. Despite advances in cytoreductive surgery and chemotherapy, approximately 70–80% of patients with advanced ovarian cancer experience disease recurrence within several years after completion of first-line treatment, highlighting the persistent clinical challenges associated with this disease [4,5,6,7].

Paclitaxel plus carboplatin (TC) chemotherapy has been established as the standard first-line treatment for ovarian cancer, based on evidence from pivotal clinical trials including GOG111, OV-10, GOG158, and AGO studies [8,9,10,11]. In the recurrent setting, treatment strategies and prognosis are strongly influenced by the platinum-free interval (PFI). Recurrence occurring ≥6 months after completion of platinum-based chemotherapy is defined as PSROC, for which retreatment with platinum-based regimens is associated with significant clinical benefit [12]. However, prognosis worsens with each recurrence, and cumulative toxicities from repeated chemotherapy remain a major concern [13]. Therefore, the establishment of effective maintenance therapies to prolong remission duration and improve survival outcomes is of critical importance.

For patients with PSROC who achieve a response to chemotherapy, both the National Comprehensive Cancer Network (NCCN) guidelines [14] and the guidelines of the Japan Society of Gynecologic Oncology [15] recommend maintenance therapy with the anti-angiogenic agent BEV or the poly ADP-ribose polymerase (PARP) inhibitor OLA. BEV has demonstrated prolongation of PFS and OS in several phase III trials, including GOG213 [16], OCEANS [17], and MITO16B-MaNGO [18], regardless of BRCA mutation or homologous recombination deficiency (HRD) status. In contrast, OLA has shown significant clinical benefit particularly in patients with BRCA variant or HRD-positive tumors, as demonstrated in the SOLO2 [19,20] and Study 19 trials [21].

More recently, the KGOG3052 trial [22] directly compared OLA and BEV as maintenance therapy in patients with BRCA variant–positive PSROC and reported a significantly prolonged PFS in the OLA group. However, evidence directly comparing these two agents regardless of BRCA variant or HRD status remains limited, and optimal treatment selection in real-world clinical practice remains controversial. Furthermore, OLA is frequently associated with adverse events that necessitate dose interruption or reduction. Although dose reduction is commonly implemented to mitigate severe toxicities, evidence regarding the clinical impact of OLA dose reduction on treatment efficacy and survival outcomes remains insufficient.

In this study, we conducted a retrospective multicenter analysis to compare the efficacy and safety of BEV and OLA maintenance therapy in patients with PSROC, irrespective of BRCA variant and HRD status, with an exploratory evaluation of olaparib dose reduction in real-world practice. The aim of this study was to clarify the comparative clinical outcomes of BEV and OLA maintenance therapy and to explore whether clinically necessary OLA dose reduction appeared to compromise treatment feasibility or outcome in routine care.

## 2. Materials and Methods

### 2.1. Patients

A total of 101 patients were included who were newly diagnosed with platinum-sensitive recurrent ovarian, fallopian tube, or primary peritoneal cancer between 1 April 2018, and 30 November 2025, at Iwate Medical University Hospital, Hachinohe Red Cross Hospital, or Iwate Prefectural Central Hospital. All patients received platinum-based chemotherapy at first recurrence, achieved an objective response, and subsequently underwent maintenance therapy.

Patients were classified into three groups according to the maintenance regimen: 34 patients received BEV, 31 received standard-dose OLA, and 36 received dose-reduced OLA.

### 2.2. Treatments

At first platinum-sensitive recurrence, patients received one of the following platinum-based chemotherapy regimens: paclitaxel plus carboplatin (TC; paclitaxel 175 mg/m^2^ on Day 1 and carboplatin AUC 5 on Day 1, every 3 weeks), paclitaxel plus cisplatin (TP; paclitaxel 175 mg/m^2^ on Day 1 and cisplatin 50 mg/m^2^ on Day 1, every 3 weeks), docetaxel plus carboplatin (DC; docetaxel 60 mg/m^2^ on Day 1 and carboplatin AUC 5 on Day 1, every 3 weeks), gemcitabine plus carboplatin (GC; gemcitabine 800 mg/m^2^ on Days 1 and 8 and carboplatin AUC 5 on Day 1, every 3 weeks), pegylated liposomal doxorubicin plus carboplatin (PLDC; PLD 30 mg/m^2^ on Day 1 and carboplatin AUC 5 on Day 1, every 4 weeks), irinotecan plus cisplatin (CPT-P; irinotecan 60 mg/m^2^ on Days 1, 8, and 15 and cisplatin 60 mg/m^2^ on Day 1, every 4 weeks), or paclitaxel plus nedaplatin (paclitaxel 175 mg/m^2^ and nedaplatin 80 mg/m^2^, every 4 weeks).

When BEV was combined with chemotherapy, it was administered at a dose of 15 mg/kg every 3 weeks according to the institutional bevacizumab administration criteria for ovarian cancer [23]. BEV maintenance therapy was continued using the same dosing schedule for up to 21 cycles. OLA was initiated at a total daily dose of 600 mg. Dose interruption or reduction generally followed prior clinical trial practice, including SOLO2, according to the type and severity of adverse events. OLA maintenance therapy was continued until documented disease progression [24].

Across the three participating hospitals, platinum-based doublet chemotherapy with or without BEV followed by maintenance therapy was the standard practical approach for first platinum-sensitive recurrence. However, treatment selection was not fully standardized and was individualized according to prior maintenance exposure, biomarker status when available, treatment response, tolerability, organ function, and toxicity risk.

For patients receiving maintenance therapy for the first time, treatment selection was guided by biomarker status: OLA was preferentially selected for patients with BRCA variants, BEV for homologous recombination–proficient (HRP) tumors, and either agent was permitted for patients with HRD based on clinical considerations. In patients with a history of prior maintenance therapy, treatment selection was based on the ESGO–ESMO guidelines [25], with switching of the maintenance agent generally recommended. Re-treatment with BEV was allowed in patients who had previously received more than 21 cycles of BEV, and re-treatment with OLA was permitted in patients with BRCA variants who had received OLA for at least 12 months during prior maintenance therapy.

### 2.3. Endpoints/Variables

The primary endpoint of this study was PFS. Secondary endpoints included OS and treatment-related adverse events. Tumor response and disease progression were assessed according to the Response Evaluation Criteria in Solid Tumors (RECIST) version 1.1 [26]. Adverse events and treatment-related toxicities were evaluated and graded based on the Common Terminology Criteria for Adverse Events (CTCAE) version 5.0, Japanese Clinical Oncology Group (JCOG) edition [27]. Adverse events were recorded cumulatively across chemotherapy and subsequent maintenance therapy.

### 2.4. Statistical Analysis

The primary analysis compared progression-free survival between BEV and all OLA-treated patients (combined OLA). The comparison between standard-dose and dose-reduced OLA (OLA-S vs. OLA-R) was conducted as an exploratory, descriptive analysis because of the inherent immortal time bias.

The data cutoff date was 30 November 2025. The platinum-free interval (PFI) was defined as the time from the last dose of the previous platinum-based chemotherapy to the diagnosis of recurrence [28]. PFS and OS were calculated from the initiation of treatment for recurrent disease to the date of second progression, death from any cause, or last follow-up, whichever occurred first.

Survival curves for PFS and OS were generated using the Kaplan–Meier method and compared between groups using the log-rank test. Comparisons of patient characteristics and adverse events among groups were performed using the chi-squared test or the Kruskal–Wallis test, as appropriate.

To evaluate the association between clinical variables and survival outcomes, univariate Cox proportional hazards models were first applied to estimate hazard ratios (HRs) and 95% confidence intervals (CIs). Exploratory multivariable Cox proportional hazards models were then constructed using treatment, PFI, histology, and tumor response as prespecified clinically relevant variables. Because this was a small three-arm retrospective cohort, propensity score matching was not feasible without substantial loss of analyzable cases. Inverse Probability of Treatment Weighting (IPTW) was performed as a sensitivity-oriented exploratory analysis; however, because substantial post-weighting imbalance remained across several key covariates, the weighted estimates were not considered sufficiently robust and were therefore not adopted as the primary analytic approach. Therefore, multivariable Cox regression was retained as the principal adjusted analysis, and the study was interpreted as exploratory and hypothesis-generating. An exploratory analysis of OLA dose reduction patterns and relative dose intensity (RDI) was also performed.

All statistical analyses were conducted using EZR (Easy R), version 1.54 (Saitama Medical Center, Jichi Medical University), and a two-sided *p*-value of <0.05 was considered statistically significant [29].

## 3. Results

### 3.1. Patient Characteristics

Patient characteristics are summarized in Table 1. The median age was 62 years (range, 33–82) in the BEV group, 59 years (46–74) in the OLA-S group, and 63 years (40–80) in the OLA-R group. Regarding disease stage 8/26, 8/23, and 4/32 patients were classified as I–II/III–IV, respectively. Serous/endometrioid-clear cell/mucinous/other histological subtypes were classified into proportions of 28/5/1, 27/3/1, and 34/2/0, respectively. The number of HRD-positive cases was found to be 5, 4, and 0, while there were 4, 5, and 2 BRCA mutation-positive cases in the BEV, OLA-S, and OLA-R groups, respectively. Regarding prior maintenance therapy, BEV had been administered in 6, 15, and 15 patients; OLA in 12, 3, and 3 patients; and BEV plus OLA in 1, 0, and 0 patients, respectively. The median PFI was 17.5 months (range, 6–140), 19 months (6–114), and 21 months (6–96), respectively. Chemotherapy regimens used before maintenance therapy included TC (±BEV) in 27, 22, and 28 patients; TP (±BEV) in 1, 3, and 0 patients; DC (±BEV) in 1 patient in each group; PLDC (±BEV) in 5 patients in each group; and other regimens in 0, 0, and 2 patients, respectively. Tumor response to chemotherapy immediately prior to maintenance therapy consisted of complete response (CR) in 7, 10, and 11 patients, and partial response (PR) in 27, 21, and 25 patients, respectively. No significant difference in response rate was observed among the groups (*p* = 0.515).

### 3.2. Survival Analysis

**Figure 1:**
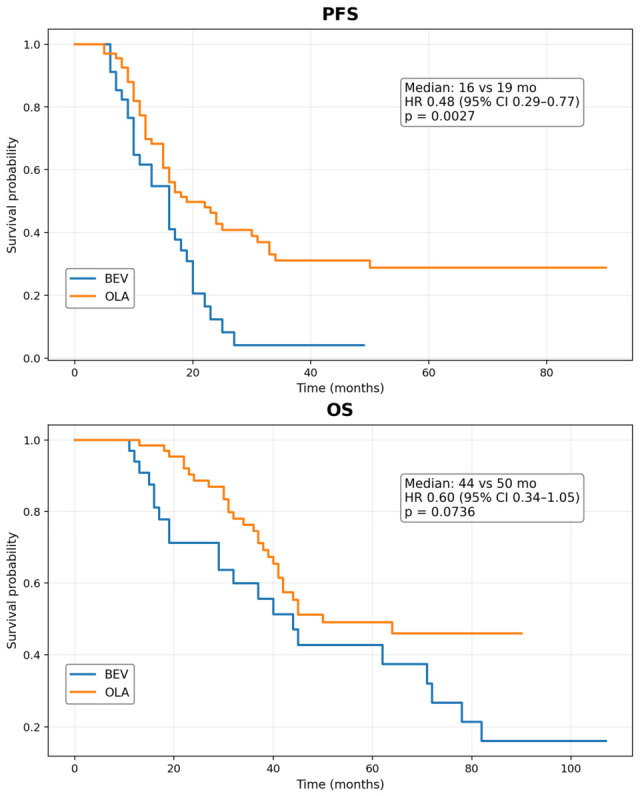
Kaplan–Meier curves for BEV versus combined OLA groups. The median PFS was 16 months (95% CI 10–19) in the BEV group and 19 months (95% CI 15–31) in the OLA group. The hazard ratio was 0.48 (95% CI 0.29–0.77; *p* = 0.0027). The median OS was 44 months in the BEV group and 50 months in the OLA group. The hazard ratio was 0.60 (95% CI 0.34–1.05; *p* = 0.0736).

The median follow-up duration was 31 months (range = 10–107) in the BEV group and 41 months (7–90) in the overall OLA group. Within the OLA cohort, the median follow-up was 32 months (7–88) in the standard-dose group and 41.5 months (22–90) in the dose-reduced group. Kaplan–Meier survival curves for PFS and OS are shown in Figure 1. In the primary comparison of all OLA-treated patients versus BEV, the median PFS was 16 months in the BEV group and 19 months in the combined OLA group, and OLA was associated with longer PFS than BEV (HR 0.48, 95% CI 0.29–0.77; *p* = 0.0027). In the exploratory comparison between OLA-S and OLA-R, the reduced-dose group showed numerically longer PFS; however, this finding is inherently confounded by immortal time bias. By contrast, median OS was 44 months in the BEV group and 50 months in the combined OLA group, but the OS difference did not reach statistical significance (HR 0.60, 95% CI 0.34–1.05; *p* = 0.0736).

**Figure 2:**
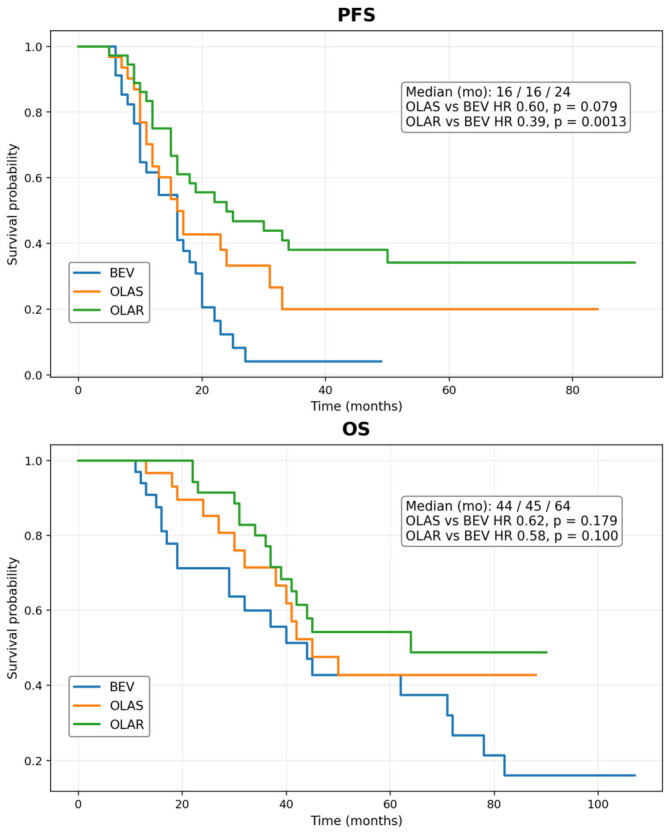
Kaplan–Meier curves for the three treatment groups. Kaplan–Meier curves for the three treatment groups. Key results are displayed within each panel. Median PFS was 16, 16, and 24 months in the BEV, OLA-S, and OLA-R groups, respectively. Compared with BEV, OLA-R was associated with significantly longer PFS (HR 0.39, 95% CI 0.22–0.69; *p* = 0.0013), whereas OLA-S was not significantly different (HR 0.60, 95% CI 0.34–1.06; *p* = 0.079). Median OS was 44, 45, and 64 months, respectively, and no statistically significant OS difference was observed among the three groups.

Figure 2 presents PFS and OS stratified by OLA dose intensity. The median PFS was 16 months in the OLA-S group and 24 months in the OLA-R group. Compared with the BEV group, the hazard ratio for PFS was 0.60 (95% CI 0.34–1.06; *p* = 0.079) in the OLA-S group and 0.39 (95% CI 0.22–0.69; *p* = 0.0013) in the OLA-R group. The median OS values were 44 months in the BEV group, 45 months in the OLA-S group, and 64 months in the OLA-R group; however, neither OLA group showed a statistically significant OS advantage over BEV (OLA-S: HR 0.62, 95% CI 0.31–1.24; *p* = 0.179; OLA-R: HR 0.58, 95% CI 0.31–1.11; *p* = 0.100). Because patients in the dose-reduced group had to remain on treatment long enough to undergo dose reduction, these subgroup comparisons are exploratory and subject to immortal time bias.

Exploratory Cox analyses are shown in Table 2 and Table 3. In multivariable analysis for PFS, PFI ≥ 12 months (HR 0.51, 95% CI 0.30–0.88; *p* = 0.015) and OLA-R versus BEV (HR 0.44, 95% CI 0.24–0.80; *p* = 0.007) were independently associated with longer PFS, whereas OLA-S versus BEV, histology, and tumor response were not significant. In multivariable analysis for OS, treatment group, histology, and tumor response were not independently associated with outcome; PFI ≥ 12 months remained significantly associated with improved OS (HR 0.40, 95% CI 0.22–0.73; *p* = 0.003).

### 3.3. Adverse Events

Major adverse events are summarized in Table 4. These adverse events represent cumulative toxicities observed during chemotherapy and subsequent maintenance therapy. Grade ≥ 3 hematologic toxicities in the BEV, OLA-S, and OLA-R groups included leukopenia in 8, 4, and 8 patients, neutropenia in 10, 9, and 18 patients, anemia in 4, 3, and 18 patients, and thrombocytopenia in 4, 1, and 2 patients, respectively. A statistically significant difference was observed only for anemia, which was more frequent in the OLA-R group (*p* = 0.010). Febrile neutropenia occurred in two patients in the BEV group only.

Grade ≥ 3 non-hematologic toxicities included nausea in 2, 2, and 5 patients, peripheral neuropathy in 4, 2, and 1 patients and fatigue in 2 patients in each group. Diarrhea was observed in one patient in the OLA-S group, and anorexia in one patient in the BEV group. Hypertension occurred in 8 patients in the BEV group and 3 patients in the OLA-S group but was not observed in the OLA-R group, showing a significant intergroup difference (*p* = 0.005). Proteinuria was observed in 4 patients in the BEV group and 2 patients in the OLA-S group. Carboplatin allergy was observed only in the OLA-R group (2 patients).

**Figure 3:**
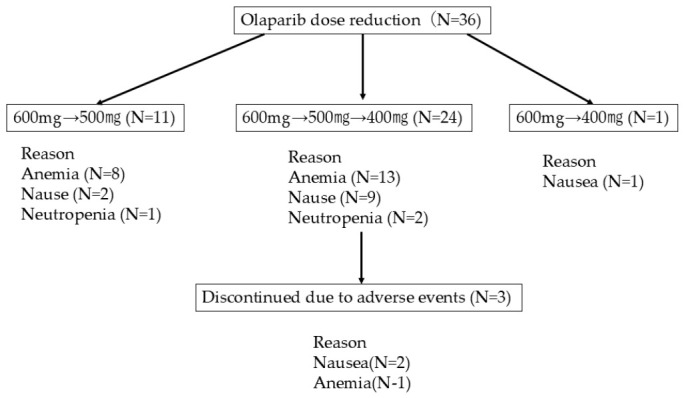
CONSORT style flowchart illustrating OLA dose modification. The diagram summarizes dose reduction patterns among 36 patients in the dose-reduced OLA group. Three reduction pathways were observed: 600 mg to 500 mg (n = 11), 600 mg to 500 mg to 400 mg (n = 24), and direct reduction from 600 mg to 400 mg (n = 1, grade 3 nausea). Three patients discontinued treatment due to adverse events after dose reduction (nausea, n = 2, anemia, n = 1).

Among patients in the OLA-R group, the median time to the first dose reduction was 2 months (95% CI 1–4), and the most common documented reasons were anemia in 22 patients (61.1%), nausea in 11 patients (30.6%), and neutropenia in 3 patients (8.3%). In addition, three patients discontinued OLA due to adverse events, including two because of nausea and one because of anemia. The direct reduction from 600 mg/day to 400 mg/day occurred in one patient with grade 3 nausea. Dose modification was generally managed according to prior clinical trial practice, including SOLO2. A detailed flowchart of dose reduction patterns is presented in Figure 3. The median relative dose intensity (RDI) in the OLA-R group was 70.5% (95% CI 68.0–78.0). In contrast, the median RDI in the OLA-S group was 100% (95% CI 98.0–100).

## 4. Discussion

This study focused on PSROC, a setting in which maintenance therapy is used to prolong remission after response to platinum rechallenge. In this biomarker-heterogeneous real-world cohort, OLA was associated with longer PFS than BEV, whereas OS did not differ significantly. Analyses according to OLA dose intensity were exploratory, and we do not interpret the findings as evidence that dose reduction improved efficacy.

These results are broadly consistent with the established role of PARP inhibitors as maintenance therapy in PSROC. OLA improved PFS in SOLO2 and Study 19, and a direct comparison in the BRCA-mutated population in KGOG3052 also favored OLA over BEV [19,20,21,22]. Similar maintenance activity has also been reported with other PARP inhibitors. In the NORA trial, niraparib maintenance using an individualized starting dose improved PFS compared with the placebo group and showed a favorable OS trend in the intention-to-treat population with PSROC [30]. In ARIEL3 trial, rucaparib demonstrated sustained maintenance effects in recurrent PSROC compared with the placebo group, as assessed within an analytical framework based on biomarker information, including BRCA mutations, HRD, and the intention-to-treat cohort [31]. Together, these studies support a broader maintenance role for PARP inhibitors in PSROC, although cross-trial comparisons should be interpreted cautiously. By contrast, BEV has demonstrated clinically meaningful activity in OCEANS, GOG213, and MI-TO16B-MaNGO [16,17,18]. Our findings fit within this therapeutic landscape and suggest that, in real-world biomarker-heterogeneous PSROC, OLA may provide greater PFS benefit than BEV, whereas any OS advantage remains uncertain.

At the same time, reports directly comparing OLA and BEV regardless of BRCA mutation or HRD status remain limited, and clear criteria for selecting between maintenance options have not been established in routine clinical practice. Interpretation of biomarker-related findings is also complicated in daily practice. In Japan, BRCA testing became reimbursed in 2018 and HRD testing in 2021; however, access to testing has not always been uniform, partly because of cost and incomplete patient uptake. Although Yoshihara et al. analyzed BRCA testing in a large Japanese cohort, that study was conducted within a clinical trial framework in which testing costs were not borne by patients [32]. Therefore, establishing effective maintenance strategies for patients with negative or unavailable biomarker results remains an important unmet clinical need. In our cohort, BRCA mutation and HRD status were unknown in a substantial proportion of patients, and the untested population likely included a mixture of HRD-positive and HRP cases. This limitation may have influenced treatment selection and outcomes and should be considered when interpreting the apparent benefit of OLA in this biomarker-heterogeneous population. In addition, the higher proportion of prior bevacizumab exposure in the OLA groups likely reflects real-world treatment sequencing. ESGO-ESMO guidance recommends switching maintenance strategy [25], whereas PARP rechallenge has not been routinely recommended [33]; therefore, patients previously exposed to bevacizumab were more likely to receive OLA at recurrence. This pattern may reflect a clinically plausible treatment sequence in routine practice, but it is also an important source of confounding when interpreting comparisons between groups.

The dose-reduction findings require careful interpretation. In the present study, the most consistent signal was observed for PFS rather than OS. Compared with BEV, the combined OLA cohort had longer PFS, while OS did not differ significantly. Within the three-group comparison, the OLA-R group showed the longest median PFS and remained associated with improved PFS versus BEV in exploratory multivariable analysis; however, no statistically significant OS advantage was demonstrated for either OLA subgroup. Because this pattern is susceptible to selection bias and time-dependent bias, we do not interpret it as evidence that OLA-R was superior to OLA-S. Rather, the findings suggest that clinically necessary dose adjustment did not appear to abolish therapeutic benefit. This interpretation is supported by the timing and pattern of dose modification in our cohort. The median time to first dose reduction was 2 months, indicating that many dose modifications occurred relatively early during treatment. This timing may limit, but does not remove, the potential impact of immortal time bias. In addition, the reasons for dose reduction and baseline frailty may also have influenced outcome. Therefore, the present findings should be interpreted as suggesting that clinically necessary dose adjustment did not clearly abolish benefit, rather than indicating that dose reduction itself improved prognosis.

Regarding safety, anemia occurred significantly more frequently in the OLA-R group, whereas hypertension was significantly more common in the BEV group. No unexpected safety signals emerged. These findings suggest that clinically necessary dose adjustment of OLA may help maintain treatment feasibility and treatment continuity in real-world practice without necessarily compromising therapeutic benefit, although this interpretation should remain cautious given the retrospective design of the study.

Several limitations of this study should be acknowledged. First, this was a small retrospective multicenter study with only 101 patients divided into three groups, and the analyses should therefore be considered exploratory and hypothesis-generating. Second, treatment allocation was clinician-driven and influenced by biomarker information and previous maintenance exposure, leading to baseline imbalances in diagnosis, BRCA status, and prior maintenance therapy. Third, the OLA-R subgroup is susceptible to selection bias and time-dependent bias. Fourth, propensity score matching was not feasible because of the small three-arm cohort, and although IPTW was attempted, post-weighting imbalance remained across several covariates, limiting the robustness of weighted estimates. Finally, missing BRCA/HRD data restricted biomarker-adjusted interpretation. Despite these limitations, our findings provide rare real-world comparative data on BEV and OLA in a biomarker-heterogeneous PSROC population, a setting that remains highly relevant in routine clinical practice.

## 5. Conclusions

In conclusion, in this retrospective multicenter cohort of PSROC, OLA was associated with longer PFS than BEV, with manageable toxicity. However, because of the small sample size, non-randomized treatment allocation, residual imbalance, and the possibility of time-dependent bias, these findings should be interpreted cautiously. The exploratory dose-reduction analyses should not be considered proof that dose reduction itself improves outcomes. Nevertheless, clinically necessary OLA dose reduction may remain a feasible maintenance strategy for selected patients in real-world practice without necessarily compromising therapeutic benefit. Future prospective studies incorporating time-dependent analyses and comprehensive biomarker profiling will be essential to validate these exploratory findings and clarify the clinical significance of dose-adjusted OLA.

## Figures and Tables

**Table 1:** Patient characteristics.

		BEV (N = 34)	Standard-Dose OLA (N = 31)	Dose-Reduced OLA (N = 36)	*p* Value
Age	Median, Range	62 (33–82)	59 (46–74)	63 (40–80)	0.167 **
Diagnosis	Ovarian	20	18	33	0.001 *
	Fallopian Tube	5	7	2	
	Peritoneal	9	6	1	
Stage	I∼II	8	8	4	0.256 *
	III∼IV	26	23	32	
Histology	Serous/ Endometrioid	28	27	34	0.576 *
	Clear/Mucinous	5	3	2	
	Other	1	1	0	
HRD status	Positive	5	4	0	0.119 *
	Negative	4	4	2	
	Unknown	25	23	34	
BRCA variant	Positive	4	5	2	0.041 *
	Negative	12	4	5	
	Unknown	18	22	29	
Prior maintenance therapy	BEV	6	15	15	0.014 *
	OLA	12	3	3	
	BEV + OLA	1	0	0	
	No treatment	15	13	18	
PFI	Median, Range	17.5 (6–140)	19 (6–114)	21 (6–96)	0.310 **
Chemotherapy regimen before maintenance	TC (+BEV)	27	22	28	0.441 *
	TP (+BEV)	1	3	0	
	DC (+BEV)	1	1	1	
	PLDC (+BEV)	5	5	5	
	Other		0	2	
Anti-tumor response	CR	7	10	11	0.515 *
	PR	27	21	25	

BEV: bevacizumab, OLA: olaparib, HRD: homologous recombination deficiency, PFI: platinum-free interval, TC: paclitaxel + carboplatin, TP: paclitaxel + cisplatin, DC: docetaxel + carboplatin, PLDC: pegylated liposomal doxorubicin + carboplatin, CR: complete response, PR: partial response, * chi-squared test, ** Kruskal–Wallis-test.

**Table 2:** Univariable and multivariable Cox analyses of selected factors associated with PFS.

**PFS Cox Proportional Hazards Model (Univariable Analysis)**				
	**Category**	**HR**	**95% CI**	***p*** **Value**
Treatment	BEV/OLA-S	0.60	0.34–1.06	0.079
	BEV/OLA-R	0.39	0.22–0.69	0.001
Age	Per year	1.03	1.00–1.05	0.028
Stage	I–II/III–IV	1.39	0.76–2.53	0.288
Histology	HGSC and EM/CCC and M	1.34	0.67–2.70	0.409
PFI	<12/> 12 months	0.46	0.27–0.78	0.003
Prior maintenance	None/BEV/ PARPi/BEV + PARPi	1.32	0.94–1.85	0.105
Anti-tumor response	CR/PR	1.56	0.97–2.48	0.063
**PFS Cox Proportional Hazards Model (Multivariable Analysis)**				
	**Category**	**HR**	**95% CI**	***p*** **Value**
Treatment	BEV/OLA-S	0.68	0.37–1.22	0.196
	BEV/OLA-R	0.44	0.24–0.80	0.007
PFI	<12/> 12 months	0.51	0.30–0.88	0.015
Histology	HGSC and EM/CCC and M	0.98	0.48–2.00	0.956
Anti-tumor response	CR/PR	1.15	0.63–2.09	0.648

PFS: progression-free survival, PARP: poly ADP-ribose polymerase, PFI: platinum-free interval, BEV: bevacizumab, OLA-S: standard-dose OLA, OLA-R: dose-reduced OLA, HGSC: high grade serous carcinoma, EM: endometrioid, CCC: clear cell carcinoma, M; mucinous, CR: complete response, PR: partial response, HR: hazard ratio, 95% CI: 95% confidence interval. Multivariable models included treatment, PFI, histology, and tumor response.

**Table 3:** Univariable and multivariable Cox analyses of selected factors associated with OS.

**OS Cox Proportional Hazards Model (Univariable Analysis)**				
	**Category**	**HR**	**95% CI**	***p*** **Value**
Treatment	BEV/OLA-S	0.62	0.31–1.24	0.179
	BEV/OLA-R	0.58	0.31–1.11	0.100
Age	Per year	1.02	0.99–1.05	0.191
Stage	I–II/III–IV	1.40	0.68–2.89	0.363
Histology	HGSC and EM/CCC and M	1.35	0.57–3.19	0.499
PFI	<12/> 12 months	0.38	0.21–0.69	0.001
Prior maintenance	None/BEV/ PARPi/BEV + PARPi	1.45	0.95–2.21	0.085
Anti-tumor response	CR/PR	1.20	0.68–2.11	0.531
**OS Cox Proportional Hazards Model (Multivariable Analysis)**				
	**Category**	**HR**	**95% CI**	***p*** **Value**
Treatment	BEV/OLA-S	0.64	0.32–1.28	0.209
	BEV/OLA-R	0.59	0.30–1.15	0.120
PFI	<12/> 12 months	0.40	0.22–0.73	0.003
Histology	HGSC and EM/CCC and M	1.10	0.46–2.65	0.832
Anti-tumor response	CR/PR	0.85	0.42–1.72	0.646

OS: overall survival, PARP: poly ADP-ribose polymerase, PFI: platinum-free interval, BEV: bevacizumab, OLA-S: standard-dose OLA, OLA-R: dose-reduced OLA, HGSC: high grade serous carcinoma, EM: endometrioid, CCC: clear cell carcinoma, M; mucinous, CR: complete response, PR: partial response, HR: hazard ratio, 95% CI: 95% confidence interval. Multivariable models included treatment, PFI, histology, and tumor response.

**Table 4:** (**a**). Adverse events (hematologic toxicities). (**b**). Adverse events (non-hematologic toxicities).

**(a)**							
	**BEV (N = 34)**		**OLA-S (N = 31)**		**OLA-R (N = 36)**		***p*** Value *
	G3	G4	G3	G4	G3	G4	
Leukopenia	7	1	4	0	8	0	0.476
Neutropenia	6	4	8	1	12	6	0.126
Anemia	4	0	3	0	9	4	0.010
Thrombocytopenia	1	3	1	0	2	0	0.315
Febrile neutropenia	1	1	0	0	0	0	0.123
**(b)**							
	**BEV (N = 34)**		**OLA-S (N = 31)**		**OLA-R (N = 36)**		***p*** Value *
	G3	G4	G3	G4	G3	G4	
Nausea	2	0	2	0	5	0	0.434
Neuropathy	4	0	2	0	1	0	0.307
Diarrhea	0	0	1	0	0	0	0.320
Fatigue	2	0	2	0	2	0	0.983
Appetite Loss	1	0	0	0	0	0	0.353
Hypertension	8	0	3	0	0	0	0.005
Proteinuria	4	0	1	1	0	0	0.101
CBDCA hypersensitivity	0	0	2	0	0	0	0.100

(**a**) BEV: bevacizumab, OLA: olaparib, OLA-S: standard-dose OLA, OLA-R: dose-reduced OLA, G: grade. Values are counts of grade 3 and grade 4 events, and adverse events were recorded cumulatively during chemotherapy plus maintenance therapy. * chi-squared test. (**b**) BEV: bevacizumab, CBDCA: carboplatin, OLA-S: standard-dose OLA, OLA-R: dose-reduced OLA, G: grade. Values are counts of grade 3 and grade 4 events, and adverse events were recorded cumulatively during chemotherapy plus maintenance therapy. * chi-squared test.

## Data Availability

The data presented in this study are available in this article.
